# Supplementary material for: Causes and consequences of sex-chromosome turnovers in Diptera
Source: Evol Lett. 2026 Mar 12;10(3):253–68. doi: 10.1093/evlett/qrag003 (PMC13231274; doi:10.1093/evlett/qrag003)
Supplement: qrag003_Supplemental_Files [file qrag003_supplemental_files.zip › Supplementary_Tables 4.zip!/Supplementary_Tables 4/Table_S5_AncestralX_genes_Calyptratae.pdf]

Table S5: The fate of genes ancestrally located on the X of Calyptratae. The location of genes that are on element F in both *D. melanogaster* and *S. caerulescens* in each of the Muscidae genome assemblies. When a chromosome level assembly was available, both the name of the chromosome, and its corresponding Muller element, is given. Rows highlighted in yellow correspond to putative minute X genes, rows highlighted in pink correspond to genes that were lost, and rows highlighted in blue correspond to genes that moved from the ancestral X to other Muller elements. Hdia: *Hydrotaea diabolus*, Hcyr: *Hydrotaea cyrtoneurina,* Ecya: *Eudasyphora cyanicolor*, Mlev: *Muscina levida*, Mdom: *Musca domestica*, Hirr: *Haematobia irritans*, Ptie: *Phaonia tiefii*, Pdow: *Philornis downsi*, Pdom: *Polietes domitor*, Scal: *Stomoxys calcitrans.*

Assignment	Gene	HydDiab	HydCyrt	EudCyai	MusLevi	Musca	Hornfly	PhaTief	PhilDow	PolDomi	StoCalc
miniX	FBgn0004607	B2_F	Chr6_F	Chr3_A	Chr3_A	KB859890.1	PGFW01009544.1	CAWVPJ010000055.1_F	JAHXMU010000735.1	CANDYM010000003.1_F	Chr1_D
miniX	FBgn0005558	B1_F	<NA>	Chr3_A	Chr3_A	<NA>	<NA>	Chr2_B	JAHXMU010000735.1	<NA>	Chr1_D
miniX	FBgn0005561	B1_F	<NA>	<NA>	<NA>	<NA>	<NA>	<NA>	<NA>	<NA>	<NA>
miniX	FBgn0026199	B1_F	<NA>	Chr3_A	Chr3_A	AQPM01023886.1	PGFW01006515.1	<NA>	JAHXMU010000735.1	<NA>	Chr1_D
miniX	FBgn0028996	B1_F	Chr6_F	Chr3_A	Chr3_A	AQPM01040023.1	PGFW01064458.1	Chr2_B	JAHXMU010000735.1	<NA>	Chr1_D
miniX	FBgn0039915	B1_F	<NA>	Chr3_A	Chr3_A	KB857857.1	PGFW01053295.1	<NA>	JAHXMU010000735.1	<NA>	Chr1_D
miniX	FBgn0039932	B1_F	Chr6_F	Chr3_A	Chr5_C	<NA>	<NA>	Chr5_C	JAHXMU010000026.1	<NA>	Chr1_D
miniX	FBgn0039937	B2_F	<NA>	Chr3_A	Chr3_A	KB854781.1	PGFW01034113.1	Chr4_A	JAHXMU010000231.1	<NA>	Chr1_D
miniX	FBgn0053978	B2_F	Chr6_F	Chr3_A	Chr4_D	KB857115.1	PGFW01052692.1	Chr3_D	JAHXMU010000090.1	<NA>	Chr1_D
miniX	FBgn0250819	<NA>	Chr6_F	<NA>	Chr4_D	KB862956.1	PGFW01058477.1	<NA>	JAHXMU010025114.1	Chr2_B	<NA>
lost	FBgn0002521	<NA>	<NA>	<NA>	<NA>	<NA>	<NA>	<NA>	<NA>	<NA>	<NA>
lost	FBgn0011747	<NA>	<NA>	<NA>	<NA>	<NA>	<NA>	<NA>	<NA>	<NA>	<NA>
lost	FBgn0013749	<NA>	<NA>	<NA>	<NA>	<NA>	<NA>	<NA>	<NA>	<NA>	<NA>
lost	FBgn0019650	<NA>	<NA>	<NA>	<NA>	<NA>	<NA>	<NA>	<NA>	<NA>	<NA>
lost	FBgn0039897	<NA>	<NA>	<NA>	<NA>	<NA>	<NA>	<NA>	<NA>	<NA>	<NA>
lost	FBgn0039911	<NA>	<NA>	<NA>	<NA>	<NA>	<NA>	<NA>	<NA>	<NA>	<NA>
lost	FBgn0039914	<NA>	<NA>	<NA>	<NA>	<NA>	<NA>	<NA>	<NA>	<NA>	<NA>
lost	FBgn0039916	<NA>	<NA>	<NA>	<NA>	<NA>	<NA>	<NA>	<NA>	<NA>	<NA>
lost	FBgn0039923	<NA>	<NA>	<NA>	<NA>	<NA>	<NA>	<NA>	<NA>	<NA>	<NA>
lost	FBgn0039925	<NA>	<NA>	<NA>	<NA>	<NA>	<NA>	<NA>	<NA>	<NA>	<NA>
lost	FBgn0039927	<NA>	<NA>	<NA>	<NA>	<NA>	<NA>	<NA>	<NA>	<NA>	<NA>
lost	FBgn0039928	<NA>	<NA>	<NA>	<NA>	<NA>	<NA>	<NA>	<NA>	<NA>	<NA>
lost	FBgn0039936	<NA>	<NA>	<NA>	<NA>	<NA>	<NA>	<NA>	<NA>	<NA>	<NA>
lost	FBgn0051999	<NA>	<NA>	<NA>	<NA>	<NA>	<NA>	<NA>	<NA>	<NA>	<NA>
lost	FBgn0052850	<NA>	<NA>	<NA>	<NA>	<NA>	<NA>	<NA>	<NA>	<NA>	<NA>
lost	FBgn0053481	<NA>	<NA>	<NA>	<NA>	<NA>	<NA>	<NA>	<NA>	<NA>	<NA>
lost	FBgn0087002	<NA>	<NA>	<NA>	<NA>	<NA>	<NA>	<NA>	<NA>	<NA>	<NA>
lost	FBgn0262636	<NA>	<NA>	<NA>	<NA>	<NA>	<NA>	<NA>	<NA>	<NA>	<NA>
moved	FBgn0004859	Chr3_A	Chr2_A	Chr3_A	Chr3_A	KB855946.1	PGFW01065786.1	Chr4_A	JAHXMU010025182.1	Chr3_A	Chr4_A
moved	FBgn0005666	Chr1_E	Chr1_E	Chr1_E	Chr1_E	KB858130.1	PGFW01068902.1	Chr1_E	JAHXMU010000717.1	Chr1_E	Chr2_E
moved	FBgn0011642	Chr4_D	Chr4_D	Chr2_D	Chr4_D	KB854593.1	PGFW01026465.1	Chr3_D	JAHXMU010000185.1	Chr4_D	Chr1_D
moved	FBgn0022361	<NA>	Chr2_A	Chr3_A	Chr3_A	KB856193.1	PGFW01037579.1	Chr4_A	JAHXMU010025345.1	Chr3_A	Chr4_A
moved	FBgn0024728	Chr4_D	Chr4_D	Chr2_D	Chr4_D	KB855180.1	PGFW01020429.1	Chr3_D	JAHXMU010000154.1	Chr4_D	Chr1_D
moved	FBgn0024811	Chr3_A	Chr2_A	Chr3_A	Chr3_A	KB855870.1	<NA>	Chr4_A	JAHXMU010000095.1	Chr3_A	Chr4_A
moved	FBgn0024913	<NA>	<NA>	<NA>	<NA>	<NA>	<NA>	<NA>	JAHXMU010000118.1	<NA>	<NA>
moved	FBgn0025726	Chr2_B	Chr3_B	Chr4_B	Chr2_B	KB855301.1	PGFW01003493.1	Chr2_B	JAHXMU010025818.1	Chr2_B	Chr3_B
moved	FBgn0025740	Chr4_D	Chr4_D	Chr2_D	Chr4_D	KB855416.1	<NA>	Chr3_D	JAHXMU010000074.1	Chr4_D	Chr1_D
moved	FBgn0025741	Chr1_E	Chr1_E	Chr1_E	Chr1_E	KB855140.1	PGFW01053931.1	Chr1_E	JAHXMU010000070.1	Chr1_E	Chr2_E
moved	FBgn0025936	Chr2_B	Chr3_B	Chr4_B	Chr2_B	KB855832.1	PGFW01021863.1	Chr2_B	JAHXMU010000072.1	Chr2_B	Chr3_B
moved	FBgn0026869	<NA>	<NA>	Chr4_B	<NA>	KB862249.1	<NA>	<NA>	JAHXMU010034946.1	<NA>	Chr1_D
moved	FBgn0027101	Chr1_E	Chr2_A	Chr1_E	Chr1_E	KB856097.1	PGFW01036406.1	Chr1_E	JAHXMU010000130.1	Chr3_A	Chr2_E
moved	FBgn0039889	<NA>	<NA>	<NA>	<NA>	<NA>	<NA>	<NA>	JAHXMU010024801.1	<NA>	<NA>
moved	FBgn0039890	Chr1_E	Chr1_E	Chr1_E	Chr1_E	KB855855.1	PGFW01027108.1	Chr1_E	JAHXMU010000071.1	Chr1_E	Chr2_E
moved	FBgn0039900	<NA>	<NA>	<NA>	<NA>	<NA>	PGFW01068770.1	<NA>	<NA>	<NA>	<NA>
moved	FBgn0039907	Chr5_C	Chr5_C	Chr5_C	Chr5_C	KB856509.1	PGFW01026916.1	Chr5_C	JAHXMU010000026.1	Chr5_C	Chr5_C
moved	FBgn0039908	Chr2_B	Chr3_B	Chr4_B	Chr3_A	KB856330.1	PGFW01007277.1	Chr2_B	JAHXMU010000639.1	Chr2_B	Chr3_B
moved	FBgn0039909	Chr3_A	Chr2_A	Chr3_A	Chr3_A	KB855472.1	PGFW01006048.1	Chr4_A	JAHXMU010000444.1	Chr3_A	Chr4_A
moved	FBgn0039920	Chr2_B	Chr3_B	Chr4_B	Chr2_B	KB856518.1	PGFW01003819.1	Chr2_B	JAHXMU010000634.1	Chr2_B	Chr3_B
moved	FBgn0039924	Chr3_A	Chr2_A	Chr3_A	Chr3_A	KB856650.1	PGFW01006448.1	Chr4_A	JAHXMU010000193.1	Chr3_A	Chr4_A
moved	FBgn0051997	Chr3_A	Chr2_A	Chr3_A	Chr3_A	KB855743.1	PGFW01026606.1	Chr4_A	JAHXMU010000661.1	Chr3_A	Chr4_A
moved	FBgn0051998	Chr1_E	Chr1_E	Chr1_E	Chr1_E	KB856157.1	PGFW01021250.1	Chr1_E	JAHXMU010000104.1	Chr1_E	Chr2_E
moved	FBgn0052016	<NA>	<NA>	<NA>	<NA>	<NA>	PGFW01031647.1	<NA>	JAHXMU010018062.1	<NA>	<NA>
moved	FBgn0085432	Chr5_C	Chr5_C	Chr5_C	Chr5_C	KB856763.1	PGFW01038335.1	Chr5_C	JAHXMU010026103.1	Chr5_C	Chr5_C
moved	FBgn0259214	Chr1_E	Chr1_E	Chr1_E	Chr1_E	KB855613.1	PGFW01030879.1	Chr1_E	JAHXMU010000231.1	Chr1_E	Chr2_E
moved	FBgn0259216	<NA>	<NA>	<NA>	<NA>	<NA>	PGFW01028092.1	<NA>	<NA>	<NA>	<NA>
